# The need for standardized biobanks in Kazakhstan

**DOI:** 10.5195/cajgh.2013.99

**Published:** 2014-01-24

**Authors:** Kuvat Momynaliev, Meruert Imanbekova

**Affiliations:** 1 National Center for Biotechnology, Almaty, Kazakhstan

**Keywords:** biobanks, biomedical organizations, scientific reseach, Kazakhstan

## Abstract

Biobanks are an important tool for clinical and research studies conducted on biomarkers of genetic therapy, diagnostic tests and new drugs; however, most biobanks remain incomplete and are often used without uniform standards and criteria. There is also a a lack of high-quality biological samples and many bioethical problems are often overlooked.

Currently, Kazakhstan has no standard requirements and protocols for biomedical organizations. However, .an analysis of published data shows that possibly hundreds of samples are analyzed. Therefore, an establishment of biobank with standardized requirements could create better quality research.

The National Center for Biotechnology has already started a biobank with more than 1,500 blood samples, with the ultimate goal of creating a biobank including around 10,000 blood samples of healthy volunteers, the same number of samples obtained from individuals with cardiovascular and endocrine diseases with samples stored under special conditions. The database contains demographic characteristics of donor’s medical history. Informed consent for research received from all donors. This biobank can be considered as a national resource for scientific research.

